# Multiple Factors Determine the Structure of Bacterial Communities Associated With *Aedes albopictus* Under Artificial Rearing Conditions

**DOI:** 10.3389/fmicb.2020.00605

**Published:** 2020-04-15

**Authors:** Shi Chen, Dongjing Zhang, Antonios Augustinos, Vangelis Doudoumis, Naima Bel Mokhtar, Hamidou Maiga, George Tsiamis, Kostas Bourtzis

**Affiliations:** ^1^Insect Pest Control Laboratory, Joint FAO/IAEA Division of Nuclear Techniques in Food and Agriculture, Vienna, Austria; ^2^Beneficial Insects Institute, Fujian Agriculture & Forestry University, Fuzhou, China; ^3^Key Laboratory of Tropical Disease Control of the Ministry of Education, Sun Yat-sen University–Michigan State University Joint Center of Vector Control for Tropical Diseases, Zhongshan School of Medicine, Sun Yat-sen University, Guangzhou, China; ^4^Department of Environmental Engineering, University of Patras, Agrinio, Greece; ^5^Institut de Recherche en Sciences de la Santé/Direction Régionale de l’Ouest, Bobo-Dioulasso, Burkina Faso

**Keywords:** *Aedes albopictus*, gut, microbiota, artificial rearing, SIT, culture-dependent, culture-independent

## Abstract

Insect symbionts are major manipulators of host’s behavior. Their effect on parameters such as fecundity, male mating competitiveness, and biological quality in general, can have a major influence on the effectiveness of the sterile insect technique (SIT). SIT is currently being developed and applied against human disease vectors, including *Ae. albopictus*, as an environment-friendly method of population suppression, therefore there is a renewed interest on both the characterization of gut microbiota and their exploitation in artificial rearing. In the present study, bacterial communities of eggs, larvae, and adults (both males and females) of artificially reared *Ae. albopictus*, were characterized using both culture-dependent and culture-independent approaches. Mosquito-associated bacteria corresponding to thirteen and five bacteria genera were isolated from the larval food and the sugar solution (adult food), respectively. The symbiont community of the females was affected by the provision of a blood meal. *Pseudomonas* and *Enterobacter* were either introduced or enhanced with the blood meal, whereas *Serratia* were relatively stable during the adult stage of females. Maintenance of these taxa in female guts is probably related with blood digestion. Gut-associated microbiota of males and females were different, starting early after emergence and continuing in older stages. Our results indicate that eggs contained bacteria from more than fifteen genera including *Bacillus*, *Chryseobacterium*, and *Escherichia–Shigella*, which were also main components of gut microbiota of female adults before and after blood feeding, indicating potential transmission among generations. Our results provided a thorough study of the egg- and gut-associated bacteria of artificially reared *Ae. albopictus*, which can be important for further studies using probiotic bacteria to improve the effectiveness of mosquito artificial rearing and SIT applications.

## Introduction

The tiger mosquito, *Aedes albopictus*, is known to carry dengue, chikungunya, and Zika viruses. Together with its closely related species *Aedes aegypti* they represent, in addition to significant biting nuisance, a serious health threat to more than three billion people in over 120 countries (Brady et al., 2012; WHO, 2017). The population control of these mosquito species is challenging due to the inefficiency of current conventional control methods, which are largely based on the removal of breeding sites and use of insecticides (Bourtzis et al., 2016; Pang et al., 2017; Flores and O’Neill, 2018). Several novel population suppression methods have been developed against *Aedes* species and some of them, including the sterile insect technique (SIT), the incompatible insect technique (IIT), the combined SIT/IIT and the Release of Insects carrying Dominant Lethals (RIDL), are currently being tested in small scale pilot trials (Bourtzis et al., 2016; Flores and O’Neill, 2018; Zheng et al., 2019).

All these methods should ideally be used as a component in area-wide integrated pest management programs (AW-IPM) (Bourtzis et al., 2016; Bouyer and Marois, 2018). They all depend on the mass rearing, sex separation, sterilization (or lethality), handling, packing, transportation and continuous release, at overflooding ratios, of males in the field to suppress a target population (Lees et al., 2015; Bourtzis et al., 2016). So, large production of high-quality males is required since they should be released at large numbers and should also be able to compete with wild males for mating with wild females. However, the mass rearing process, handling, packing, transportation and release as well as the irradiation (SIT), the infection with a bacterial symbiont such as *Wolbachia* (IIT or combined SIT/IIT) or the insertion of a transgene (RIDL) may have a negative effect in the life history traits of the mass reared insect strain and the overall biological quality of the released males (Lees et al., 2015; Bourtzis et al., 2016).

Many bacterial species have established long evolutionary and intimate symbiotic associations with insects affecting many aspects of their biology, ecology and evolution including nutrition, metabolism, immune function, physiology as well as reproduction and behavior (Dillon and Dillon, 2004; Bourtzis and Miller, 2009; Zchori-Fein and Bourtzis, 2011; Engel and Moran, 2013). There have been several studies which have focused on the characterization of the bacterial communities associated with insects, their role in the host biology as well as their potential exploitation to develop and/or enhance novel strategies to control populations of pests and disease vectors including mosquitoes (Douglas, 2015; Berasategui et al., 2016; Arora and Douglas, 2017; Flores and O’Neill, 2018).

As previous studies in fruit flies have shown, the biological quality and the overall ecological fitness of the insects highly depends on their associated bacterial and other microbial partners (Shelly and McInnis, 2003; Niyazi et al., 2004; Ben-Yosef et al., 2008; Ben Ami et al., 2010; Gavriel et al., 2011; Storelli et al., 2011; Blum et al., 2013; Hamden et al., 2013; Sacchetti et al., 2014; Augustinos et al., 2015; Kyritsis et al., 2017; Cai et al., 2018; Khaeso et al., 2018; Akami et al., 2019). Therefore, insect symbiotic bacterial species can be used as probiotics to improve key parameters of population suppression strategies, including the productivity of mass reared strains as well as the mating performance and longevity of sterile males (Niyazi et al., 2004; Ben Ami et al., 2010; Gavriel et al., 2010; Yuval et al., 2013; Augustinos et al., 2015; Kyritsis et al., 2017; Cai et al., 2018).

There is an increasing interest in the structure of mosquito-associated microbiota as well as the interactions between the host and the microbes (for recent reviews see Guegan et al., 2018; Strand, 2018; Scolari et al., 2019), due to the role of the associated microbes in the biology of their hosts and the immunity and pathogen interference. The latter is critical for the development and application of novel population suppression and population modification strategies against major mosquito vector species (Flores and O’Neill, 2018; Guegan et al., 2018). Many studies have investigated the role of the environment and the breeding sites on microbial acquisition in *Aedes*, *Anopheles*, and *Culex* mosquitoes and have shown that there is a clear overlap of bacterial composition between mosquito species, developmental stages, and habitats (for a recent review see Guegan et al., 2018). In addition, the concept of mosquito core- and pan-microbiota has been investigated and recent data clearly indicate that environmental factors and food resources play a major role in the environmental bacterial acquisition and this may determine key biological properties of the mosquito hosts (Pastoris et al., 1989; Lindh et al., 2005; Rani et al., 2009; Wang et al., 2011, 2018; Minard et al., 2013a, b; Guegan et al., 2018). Sex, female size, and genetic diversity have been shown to affect the associated microbiota in *Ae. albopictus* with the overall bacterial diversity of both *Ae. albopictus* and *Ae. koreicus* being significantly lower in recently invaded regions (Minard et al., 2018; Rosso et al., 2018).

Interestingly, some bacterial species (such as *Wolbachia, Asaia*, and *Elizabethkingia*) may have interstadial transmission, for example from pupae to adults or via maternal transmission (Kittayapong et al., 2002; Favia et al., 2007; Akhouayri et al., 2013; Ngwa et al., 2013). Differences in the bacterial diversity between males and females have been partly attributed to differences in the flight dispersal and blood feeding (Foster, 1995; Demaio et al., 1996; Pumpuni et al., 1996; Zayed and Bream, 2003; Gusmao et al., 2010; Kumar et al., 2010; Benoit et al., 2011; Gaio et al., 2011; Wang et al., 2011; Zouache et al., 2011). A number of studies has also shown that mosquito-associated bacterial species may affect both metabolism and life history traits including blood and sugar digestion, supply of vitamins and amino acids, body size, oviposition site choice and egg production, longevity, sex ratio, larval development as well as virus dissemination (Chouaia et al., 2012; Mitraka et al., 2013; Sharma et al., 2013; Coon et al., 2014, 2016a,b, 2017; Muturi et al., 2016; Dickson et al., 2017; Guegan et al., 2018).

The Insect Pest Control Laboratory of the Joint FAO/IAEA Division of Nuclear Applications in Food and Agriculture has been developing the SIT-based approaches for the population control of *Ae. albopictus* and guidelines for mass-rearing laboratory populations of insects that are targets for SIT. Given the importance of the gut-associated microbiota for the rearing process and the overall biological quality of artificially produced insects for SIT-based applications, the present study focused on the characterization of the gut-associated bacterial species of a laboratory strain of *Ae. albopictus* under artificial rearing conditions for potential SIT-based applications. The characterization was performed using culture-dependent and culture-independent approaches, throughout the mosquito developmental stages (egg, larva and adult), at different adult ages (both young and old males and females) and female feeding regimes (sugar or blood). The density levels of key bacterial partners were assessed, and the overall findings are discussed from an applied perspective towards the use of SIT-based approaches for the population suppression of this major mosquito vector species.

## Materials and Methods

### Mosquito Strains and Maintenance

The experiments were conducted at the Joint FAO/IAEA Insect Pest Control Laboratory (hereafter IPCL), Seibersdorf, Austria. *Aedes albopictus* wild type strain (Guangzhou, China), known as GUA strain (Zhang et al., 2015) at F13 generation, was used in these experiments. Egg hatching, larval rearing, and adult maintenance were carried out as previously reported (Zhang et al., 2015). Laboratory-reared cyclic colony was maintained under at 26 ± 1°C, 60 ± 10% RH with a photoperiod of 12: 12 h (L: D).

### Sample Collection and Dissection

Blood meals were provided to the GUA females aged 7- to 9-days post emergence. Two days after the blood meal, a plastic 250-ml beaker containing 100 mL sterilized deionized water and a strip of sterilized white filter paper (white creped papers IF C140, Industrial Filtro S.r.l., Cologno Monzese, Italy) were placed in the cage (30 × 30 × 30 cm, BugDorm 1, MegaView, Taichung, Taiwan). Fresh eggs (less than 4 h) were collected and counted (approximately 100 for each replicate) as materials for bacterial culture. The rest of the eggs were maintained in the adult rearing room for 7 days for maturation and then hatched as previously described (Zhang et al., 2015). Larvae were fed on modified IAEA liquid larvae diet (Balestrino et al., 2014). The whole gut of the 3rd instar larvae (L3) was dissected and was used as source for the isolation of cultivable bacterial species. Pupae were collected and separated using an improved Fay-Morlan separator (Dame et al., 1974; Focks, 1980).

Male and female pupae were reared separately in sterilized deionized water and placed in cage for emergence. Non-fed less than 24 h old adults (both males and females) were collected and were used for the dissection of whole guts. The rest of the adults were supplied with 10% sucrose solution. Part of the females were offered with defibrinated pig blood (Rupert Seethaler, Vienna, Austria) in sausages (EDICAS, Girona, Spain) from 7th day to 9th day after emergence. Whole guts of blood-fed females were dissected at the 14th day after emergence to allow full digestion of blood. In parallel, whole guts of males and non-blood fed females were also collected. Ventral diverticulum and Malpighian tubules were removed from all gut samples.

Samples from seven groups, including eggs (EGG), dissected guts of larvae (LAR), up to 1-day old non-fed males (1DM), up to 1-day old non-fed females (1DF), 14 days old sucrose-fed males (14DM), 14 days old non-blood fed (sucrose-fed) females (NBF), and 14 days old blood-fed females (BFF) were included in both culture-dependent and culture-independent approaches. Fresh eggs were collected with sterilized dissecting needles. Alive adults which had been anesthetized at 4°C and alive larvae were surface disinfected by dipping in 70% ethanol for 1 min, placed into 1 × PBS (phosphate buffer saline) for rinsing, and then dissected in PBS under a binocular microscope with sterilized needles to get whole guts under aseptic conditions. One hundred fresh eggs or 5 whole guts were pooled to create one sample (replicate).

### Culture-Dependent Approaches

For the isolation of cultivable gut bacteria, samples were collected in 1.5 mL Eppendorf tubes with 200 μL sterile LB medium (Invitrogen). Samples were mechanically crushed using sterile pestles and 800 μL LB were added to make a total volume of 1,000 μL. The homogenate was serially diluted (from 10^0^ to 10^–4^) and plated on three types of agar media, one non-selective (LB Agar, Life technologies) and two types of selective media, Chromocult Agar ES and XLD Agar (Merck Millipore). Three replicates with 100 μL solution were used for each medium. Plates were incubated in incubator (IPP110, Memmert, United States) at 26°C for 16 h (or until bacterial colonies were visible but incubation did not exceed 48 h). From the dilution series, those plates with 10 to 300 bacterial colonies were used and the number of colony-forming units (CFU) in the original solution was calculated.

Appropriate controls were also prepared, including (a) EGG-liquid: one hundred eggs were washed in 200 μL LB medium by vortex and gently centrifuged to get 100 μL supernatant as potential source of bacteria; (b) Larval food: this control was prepared with larvae diet (30 mL/L) which was maintained in a rearing room for 48 h and (c) Sugar solution: this was collected from both females’ and males’ cages at the 14th day post emergence.

Twenty bacterial isolates for each sample treatment and 12 for each control sample (eggs, larval food or sugar solution) were selected based on bacterial colony characteristics such as color, size, shape, opacity, margin, elevation and viscosity. Three rounds of streaking and isolation were performed on the corresponding medium to ensure that the bacterial isolates were pure cultures. Purified isolates were cultivated in LB medium at 26°C for 16–20 h, and then stored in 25% glycerol at −80°C.

### Colony Characterization Using 16S *rRNA* Gene-Based RFLP Assay

PCR was performed on 1 μL fresh culture liquid using 2 × Taq mix (Qiagen) and the universal 16S *rRNA* gene primers 27F/1492R (Edwards et al., 1989; Weisburg et al., 1991) with MJ Research Tetrad PTC-225 Thermal Cycler (GMI, United States). The PCR cycling conditions were template denaturation at 95°C for 10 min followed by 35 cycles of denaturation at 95°C for 45 s, primer annealing at 55°C for 1 min, and primer extension at 72°C for 2 min; a final extension step of 10 min at 72°C was also included. In case that amplification failed, PCR was carried out again with DNA extracted using Dneasy Blood and Tissue Kit (Qiagen) after lysozyme (SIGMA) digestion (resuspended in the lysis buffer with 10 mg/mL lysozyme and digested at 37°C for 2 h). Part of each PCR reaction (5/50 μL) was electrophoresed on 1.5% agarose gels, and all amplicons of the expected size were individually digested with restriction endonucleases *Taq*I, *Eco*RI, *Hae*III and *Rsa*I (Thermo) according to manufacturer’s suggestions. Specifically, 5–15 μL per amplicon were digested, using 2 μL 10 × buffer and 3–5 μL enzyme, in a final volume of 20 μL. Reactions were kept for 3–4 h at 37°C, then inactivated at 80°C and electrophoresed on 2% agarose gels. In case the selected bacterial isolates with different colony morphology characteristics were found to present quite similar RFLP patterns for all 4 enzymes, digestion with an additional restriction enzyme (*Asu*II, *Mfe*I or *Hga*I, Thermo) was included for its genotyping or their 16S *rRNA* gene was sequenced double stranded (see below).

### 16S *rRNA* Gene Sequencing and Data Analyzing

From each bacterial group and taking into consideration the RFLP pattern and colony morphology, 2–7 bacterial isolates were selected for sequencing of almost the entire 16S *rRNA* gene. PCR products were purified using the High Pure PCR product purification kit (Roche, Germany) and sequenced double stranded (VBC, Austria by using the 27F-1492R initial primer set and 4 internal primers: 519R, 596F, 960R and 1114F (Reed et al., 2002).

All 16S *rRNA* gene sequences were assembled in Sequencher 4.14, aligned with Clustal X 1.83 and BioEdit 7.01. Isolates with identical sequences were recognized with a different number. Sequences were examined for chimeras using the DECIPHER’s web tool^1^ and USEARCH 6.0^2^. Taxonomy assignment was performed using BLASTN^3^ and the closest relative was assigned using RDP classifier^4^. Alignment of sequences was carried out using MUSCLE (Edgar, 2004). A phylogenetic tree, based on the distance matrix method, was constructed using the software package Geneious 8. Evolutionary distances were calculated using the Jukes-Cantor model, and topology was inferred using the “neighbor-joining” method. A phylogenetic tree calculated by maximum parsimony, using the PAUP phylogenetic package, was also generated. Sequences with 1180 bp length were used for tree constructions.

### Culture-Independent Approaches-Next Generation Sequencing (NGS) and Statistical Analysis

Eggs and guts were mechanically crushed using sterile pestles in liquid nitrogen. DNA was extracted following the protocol of Dneasy Blood and Tissue Kit. If required, DNA was concentrated into >15 ng/μL, and three replicates per sample were prepared and sent for 16S *rRNA* gene sequencing using the MiSeq Illumina platform to the IMGM Laboratories GmbH (Martinsried, Germany) targeting two different regions with primers U341F (5′-CCTACGGGRSGCAGCAG-3′) and 805R (5′-GTGCCAGCMGCCGCGGTAA-3′) and 909F (5′-ACTCAAAKGAATWGACGG-3′) and 1391R (5′-GACGGGCGGTGWGTRCA-3′). The 16S *rRNA* gene sequences reported in this study have been deposited in NCBI under Bioproject number PRJNA575054, while the Sanger generated sequences have been deposited under accession numbers MN540103 to MN540125.

De-multiplexing and conversion to FASTQ format was performed using Qiime 1.9.1 (Caporaso et al., 2010b). Pair-end reads were assembled, trimmed and corrected for error using PandaSeq (Masella et al., 2012). Unassembled reads and reads outside the range of 440 to 450 bp once assembled were discarded. All subsequent analyses were conducted in QIIME version 1.9.1 (Caporaso et al., 2010b). Sequences were clustered into Operational Taxonomic Units (OTUs) using USEARCH (Edgar, 2010) by open-reference OTU picking. Chimeras were detected and omitted using the program UCHIME (Edgar et al., 2011) with the QIIME-compatible version of the SILVA 111 release database (Quast et al., 2013). The most abundant sequence was chosen as the representative for each OTU. Taxonomy was assigned to representative sequences by BLAST (Altschul et al., 1990) against the SILVA 111 release database (Quast et al., 2013). Representative sequences were aligned against the SILVA core reference alignment using PyNAST (Caporaso et al., 2010a). Alpha-diversity indices, as well as indices depicting the population structure, were calculated with the QIIME pipeline (Caporaso et al., 2010b) based on the rarefied OTU table at a depth of 15,000 sequences/sample (observed species, PD whole tree, chao1 and simpson reciprocal). Inter-sample diversity was calculated using Bray-Curtis distances while Principal Coordinate Analysis (PCoA) and multidimensional scaling (MDS) plot (Anderson, 2001) was performed on the resulting distance matrix. These calculations and those for alpha diversity were performed in QIIME version 1.9.1. ANOVA and Tukey–Kramer *post hoc* tests were employed to detect statistical differences (Edgar, 2010). Permutational Multivariate Analysis of Variance (PERMANOVA) analyses were applied to Bray–Curtis similarity matrices to compute similarities between groups (Anderson, 2001). Community structure differences were viewed using the constrained ordination technique CAP (Canonical Analysis of Principal Coordinates), using the CAP classification success rate and CAP trace_Q_m__’__HQ_m_ statistics, and were performed with 9999 permutations within PRIMER version 6+ (Anderson and Willis, 2003).

### Semi-Quantitative Analysis of Main Gut Bacteria Groups

qPCR-based semi-quantitative analysis was performed for some of the most relatively abundant genera (*Aeromonas*, *Asaia*, *Elizabethkingia* and *Chryseobacterium, Enterococcus*, and *Wolbachia*) detected by the 16S *rRNA* gene next generation sequencing (NGS). The qPCR analysis was carried out in three replicates on the same samples used for the NGS. It was difficult to design genus specific primers for *Elizabethkingia*, so this genus was studied as a group together with *Chryseobacterium*. The sequence of the primers and other relevant information for the qPCRs are presented in Supplementary Table 1. The *Ae. albopictus* ribosomal protein S6 (*rps*6) gene was used as control (for calibration).

A gradient PCR was initially performed to standardize the PCR conditions. PCR amplification was performed with an initial denaturation at 95°C for 5 min, followed by 35 cycles of denaturation at 95°C for 30 s, annealing at 57–64°C for 30 s, and extension at 72°C for 45 s, and kept under 72°C for 10 min. PCR products were analyzed on agarose gel electrophoresis to confirm the presence of the specific amplicon. These conditions were then used to perform qPCR analysis (Supplementary Table 1). The amplification was performed using iQ^TM^ SYBR^®^ Green Supermix (Bio-Rad, United States). The reaction mixture (15 μL) consisted of 5 ng DNA template, 2–5 pmol of each primer and 7.5 μL of 2 × Supermix. qPCR was performed with a CFX96 Touch Real-Time PCR Detection System (Bio-Rad, United States). Due to the different annealing temperatures (Supplementary Table 1), the reactions for the target genes and the housekeeping gene were put on different plates. An initial denaturation at 95°C for 2 min was followed by 40 cycles consisting of denaturation at 95°C for 10 s, at annealing temperature for 60 s. To check and confirm the quality of amplification, a melting profile was generated for the amplicon over a temperature range of 65°C to 95°C. Melting curves for each sample were analyzed after each run to check the specificity of amplification. Cq of products was calculated using CFX Manager^TM^ Software (Bio-Rad Laboratories, Inc.). Relative density of selected bacterial taxa was determined by using the 2^–ΔΔCT^ calculation method. Three biological replicates were employed for each sample (with the exception of 14DM for which only 2 replicates were used for the *Asaia* and *Chryseobacterium-Elizabethkingia* group reactions), and 2 technical replicates of qPCR were carried out for each reaction.

### Statistical Analysis of Data

Statistical analysis were carried out with JMP 14.3.0 (2018, SAS, Cary, NC, United States). The average of relative density of 2 technical replicates was used for each biological replicate. The mean and standard error (SE) of relative density was calculated, and comparisons of relative density of selected bacterial taxa between sample groups were assessed using ANOVA followed by Tukey–Kramer HSD.

## Results

### Isolation and Characterization of Cultivable Bacterial Species During the Development of *Aedes albopictus*

**FIGURE 1:**
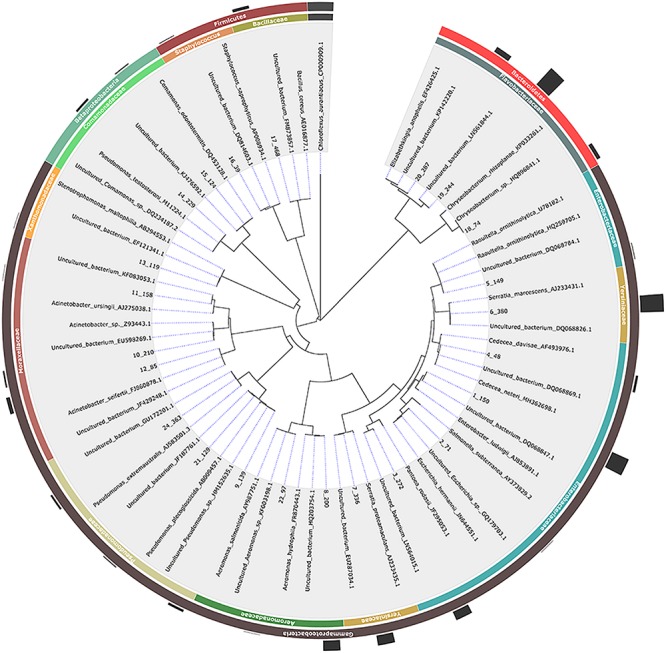
Phylogenetic relationships based on 16S *rRNA* gene sequence analysis of members of the bacterial communities associated with *Aedes albopictus*. In the cases where no published mosquito-derived sequences belonging to the same bacterial species were available, the closest hit deriving from mosquitos and the closest hit regardless of origin were included (to avoid misinterpretation of results). Evolutionary distances were calculated using the method of Jukes and Cantor and the topology was inferred using the neighbor-joining method. A representative isolate per each phylotype with their taxonomy assigned is indicated in the outer circle while the number of isolates is graphically represented. The 16S *rRNA* gene sequence of *Chloroflexus aurantiacus* was arbitrarily chosen as outgroup.

Using three different microbiological media (LB Agar, Chromocult Agar ES and XLD Agar), 462 bacterial isolates were isolated in total including 73 isolates from the three control samples. The selection of the colonies was mainly based on colony morphology since our goal was to isolate as many different bacterial species as possible for further investigation. A 16S *rRNA* gene-based PCR-RFLP assay was employed for the initial characterization and grouping of the bacterial isolates. Restriction endonucleases *Eco*RI, *Asu*II, *Mfe*I and *Hga*I exhibited up to maximum one recognition site, whereas *Taq*I, *Hae*III and *Rsa*I displayed multiple ones (Supplementary Table 2). According to the PCR-RFLP data, the bacterial isolates were placed in 27 groups. Up to two representatives from each group were selected for 16S *rRNA* gene sequencing and the sequencing data revealed the presence of 23 unique bacterial isolates, belonging to *Proteobacteria* (mainly members of Gammaproteobacteria), *Firmicutes* and *Bacteroidetes* (mainly members of Flavobacteriaceae) (Table 1 and Figure 1). Members of the *Acinetobacter*, *Bacillus*, *Cedecea*, *Chryseobacterium*, *Comamonas*, *Elizabethkingia*, *Enterobacter*, *Escherichia*, *Pseudomonas*, *Serratia*, *Staphylococcus*, and *Stenotrophomonas* genera, were retrieved from eggs and the digestive track of adult mosquitoes. Members of the *Raoultella* genus, as well as members of the *Acinetobacter* and *Pseudomonas* (but different from the ones detected in the eggs and the digestive track of adult mosquitoes), were isolated only in larval food, but not in adult mosquitoes (Table 1).

**TABLE 1:** Bacterial isolates obtained from *Ae. albopictus*; and cultivable close phylogenetic relatives (identity > 97%) from Aedes, other mosquito or other insects.

**Sample code**	**Accession Number**	**Stage/Medium (Times isolated)**	**Phylogenetic linkage**	**Phylogenetic relative and Isolation source**	**References**	**Accession No.**	**Identity(%)**
1_150	MN540103	Sugar solution from females’ cage-CC, LB, XLD (24)	*Enterobacter* sp.	*Enterobacter* sp., *Ae. albopictus* male adult, wild-caught	Zouache et al., 2011	GU726184.1	98.84
		BFF-CC (1)					
		LAR-CC, LB, XLD (24)					
		Larval food-CC, LB, XLD (19)					
2_71	MN540104	LAR-LB (1)	*Escherichia* sp.	*Klebsiella pneumoniae*, *Ae. aegypti* (Rockefeller) female adult, lab-rearing	Terenius et al., 2012	JN201948.1	97.54
				*Escherichia hermannii* (non-mosquito source)		JN644551.1	100
3_272	MN540105	BBF-CC, XLD (3)	*Enterobacter* sp.	*Enterobacter* sp., *Ae. albopictus* male adult, wild-caught	Zouache et al., 2011	GU726182.1	97.54
		1DF-CC, LB (26)					
		1DM-CC, LB (5)					
4_48	MN540106	EGG-CC (5)	*Cedecea* sp.	*Enterobacter* sp., *Ae. albopictus* male adult, wild-caught	Zouache et al., 2011	GU726183.1	97.75
				*Cedecea neteri* (non-mosquito source)		MH362698.1	100
		EGG-liquid-CC (3)					
		LAR-LB (1)					
5_149	MN540107	Larval food-XLD (2)	*Raoultella* sp.	*Serratia marcescens*, *Ae. aegypti* (Rockefeller) female adult, lab-rearing	Terenius et al., 2012	JN201947.1	97.75
				*Raoultella ornithinolytica_*		HQ259705.1	100
6_380	MN540108	NBF-CC, LB, XLD (47)	*Serratia* sp.	*Serratia marcescens*, *Ae. aegypti* (Rockefeller) female adult, lab-rearing	Terenius et al., 2012	JN201947.1	99.52
		EGG-CC, LB, XLD (41)					
7_336	MN540109	BFF-CC, LB, XLD (35)	*Serratia* sp.	*Serratia* sp., *Delia radicum* larvae, lab-rearing	Welte et al., 2016	KP836246.1	97.95
		NBF-CC (1)					
		1DF-CC (2)					
8_200	MN540110	1DF-CC, LB, XLD (5)	*Aeromonas* sp.	*Aeromonas hydrophila*, *An. maculipennis*, stage unknown, wild-caught		GU204971.1	98.64
		1DM-LB, XLD (32)					
		LAR-XLD (2)					
9_139	MN540111	Larval food-CC, LB (9)	*Pseudomonas* sp.	*Pseudomonas* sp., *Ae. albopictus*, female adult, lab-rearing	Zouache et al., 2009	FJ688378.1	97.40
10_230	MN540112	1DM-CC (6)	*Acinetobacter* sp.	*Acinetobacter beijerinckii*, *Cx. Quinquefasciatus*, female adult, wild-caught	Chandel et al., 2013	JN644620.1	97.34
11_158	MN540113	Larval food-CC (1)	*Acinetobacter* sp.	*Acinetobacter* sp., *Glossina pallidipes*, stage unknown, wild-caught		MG162615.1	98.15
12_85	MN540114	1DM-CC (3)	*Acinetobacter* sp.	*Acinetobacter* sp. *Ae. albopictus*, female adult, lab-rearing	Zouache et al., 2009	FJ688379.1	99.25
		LAR-CC, LB (11)					
13_119	MN540115	LAR-CC (1)	*Stenotrophomonas* sp.	*Stenotrophomonas maltophilia*, *An. gambiae*, female adult, lab-rearing	Lindh et al., 2008	EF426435.1	99.45
14_229	MN540116	1DF-LB (1)	*Comamonas* sp.	*Comamonas* sp., *Ae. albopictus*, female adult, lab-rearing	Zouache et al., 2009	FJ688377.1	100
		1DM-CC, LB (7)					
15_124	MN540117	LAR-CC, LB (4)	*Comamonas* sp.	*Comamonas odontotermitis*, *Odontotermes formosanus*, wild-caught	Chou et al., 2007	NR_043859.1	99.93
16_39	MN540118	EGG-LB (2)	*Staphylococcus* sp.	*Staphylococcus saprophyticus*, *Cx. Quinquefasciatus*, female adult, wild-caught	Chandel et al., 2013	JN644617.1	99.46
17_468	MN540119	Sugar solution from females’ cage-LB (6)	*Bacillus* sp.	*Bacillus cereus*, *Ae. albopictus* female adult, wild-caught	Zouache et al., 2011	GU726173.1	99.66
		Sugar solution from females’ cage-LB (2)					
		LAR-LB (1)					
		Larval food-LB (2)					
18_74	MN540120	LAR-CC, LB (14)	*Chryseobacterium* sp.	*Chryseobacterium* sp., *Cx. Quinquefasciatus*, female adult, wild-caught	Chandel et al., 2013	HQ154575.1	97.98
19_244	MN540121	BFF-LB (14)	*Elizabethkingia* sp.	*Elizabethkingia meningoseptica*, *Ae. aegypti* (Rockefeller) female adult, lab-rearing	Terenius et al., 2012	JN201943.1	97.92
		NBF-LB (12)					
		14DM-CC, LB (40)					
		EGG-CC, LB (12)					
		EGG-liquid-CC (4)					
20_287	MN540122	1DF-CC, LB (21)	*Elizabethkingia* sp.	*Elizabethkingia meningoseptica*, *Ae. aegypti* (Rockefeller) female adult, lab-rearing	Terenius et al., 2012	JN201943.1	98.34%
21_129	MN540123	Larval food-LB (3)	*Pseudomonas* sp.	*Pseudomonas* sp., *Ae. albopictus*, female adult, lab-rearing	Zouache et al., 2009	FJ688378.1	97.26
22_97	MN540124	Larval food-XLD (1)	*Aeromonas* sp.	*Aeromonas hydrophila*, *An. maculipennis*, stage unknown, wild-caught		GU204971.1	98.57
24_363	MN540125	BFF-CC, LB (7)	*Pseudomonas* sp.	*Pseudomonas ficuserectae*, *Cx. Quinquefasciatus*, female adult, wild-caught	Chandel et al., 2013	JN644593.1	97.61

Although the initial selection of colonies from all three microbiological media was based on morphological criteria and only up to 20 colonies per sample were selected, it’s still worth noting the following: (a) representatives of nine genera were isolated from LAR samples while only one bacterial strain, *Elizabethkingia* sp., was isolated from 14DM; (b) some genera were represented by multiple representatives (in some cases, they may represent different species); (c) some bacterial isolates were recovered from just a single developmental stage while others from multiple ones and (d) representatives of several bacterial genera were isolated from both mosquito and control samples including *Acinetobacter* sp., *Bacillus* sp., *Cedecea* sp., *Elizabethkingia* sp., *Enterobacter* sp., and *Pseudomonas* sp. isolates (Table 1).

### 16S *rRNA* Gene-Based Taxonomic Composition of Artificially Reared *Aedes albopictus*

The sequence data obtained for the two targeted regions of the 16S *rRNA* gene (amplified by the primer pairs U341F/805R and 909F/1391R) were compared in selected samples and no statistically significant differences were observed (Supplementary Figures 1–5). For this reason, all results presented below and in the respective figures and tables have been prepared by combining the raw data of the two 16S *rRNA* gene regions.

In total, seven samples (EGG, LAR, 1DM, 1DF, 14DM, NBF, and BFF with three replicates each) were sequenced producing 546,136 reads for bioinformatic analysis with an average of 78,014 reads per sample (Table 2). Based on alpha-diversity indices, the larval samples were the most species rich in *Ae. albopictus* (Table 2). Males and females fed with sucrose and/or blood together with the teneral females exhibited the lowest species richness and diversity indices (Table 2). Interestingly, teneral mosquitoes exhibited a sex specific differentiation (Table 2). Richness indices like Chao1 were within the range of the number of OTUs indicating, like the rarefaction analysis (Supplementary Figure 5), that sampling of each treatment has reached saturation, which was supported by the Good’s coverage index (Table 2). The dominant OTUs that were identified in the present study were classified into 5 phyla, 8 classes, 17 orders, 23 families, and 29 genera (Table 3).

**TABLE 2:** Richness and diversity estimation of the 16S *rRNA* gene libraries of *Aedes albopictus* through the amplicon sequence analysis.

**Samples**			**Number of OTUs**	**Good’s coverage**	**Number of reads**	**Species richness indices**		**Species diversity indices**	
**Stage/Sex**	**Age**	**Diet**				**Chao1**	**ACE**	**Shannon**	**Simpson**
Eggs	Eggs	BFA	114.13 ± 7.30^a^	0.998	111,107	138.37 ± 9.61^a^	137.7 ± 9.38^a^	2.57 ± 0.03^a^	0.72 ± 0.007^a^
Larva	Larva	LLD	318.13 ± 9.86^b^	0.995	62,223	362.61 ± 9.45^b^	363.9 ± 9.41^b^	5.21 ± 0.15^b^	0.91 ± 0.009^b^
Males	1 days	Teneral	142.47 ± 3.43^c^	0.998	54,555	157.50 ± 3.68^c^	157.45 ± 4.09^c^	2.64 ± 0.05^c^	0.64 ± 0.021^c^
Females	1 days	Teneral	61.20 ± 2.69^d^	0.999	82,200	72.63 ± 3.65^d^	69.97 ± 2.93^d^	0.84 ± 0.06^d^	0.17 ± 0.014^d^
Males	14 days	Sucrose	67.17 ± 7.61^d^	0.999	101,620	76.74 ± 8.10^d^	76.61 ± 7.47^d^	2.22 ± 0.16^e^	0.57 ± 0.044^e^
Females	14 days	Sucrose	61.57 ± 3.01^d^	0.998	57,246	86.67 ± 4.72^e^	81.43 ± 3.15^e^	1.59 ± 0.13^f^	0.50 ± 0.043^e^
Females	14 days	Blood	61.70 ± 2.29^d^	0.998	77,145	81.27 ± 3.54^e^	80.48 ± 3.32^e^	1.02 ± 0.02^g^	0.30 ± 0.002^f^

*For each diversity index, ANOVAs followed by the Tukey HSD test, (*p* < 0.05) were performed. Significant differences are indicated by different letters.*

**TABLE 3:** Taxonomic composition of the 16S *rRNA* gene sequencing data in the three analyzed populations.

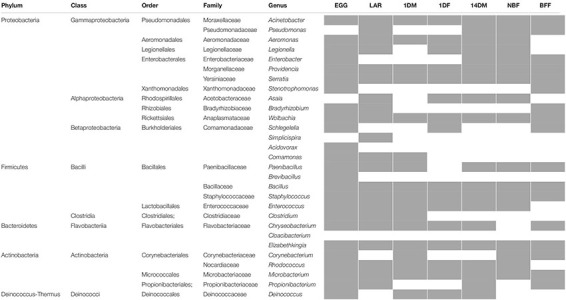

*Cells with a RA more than 1% they appear gray.*

**FIGURE 2:**
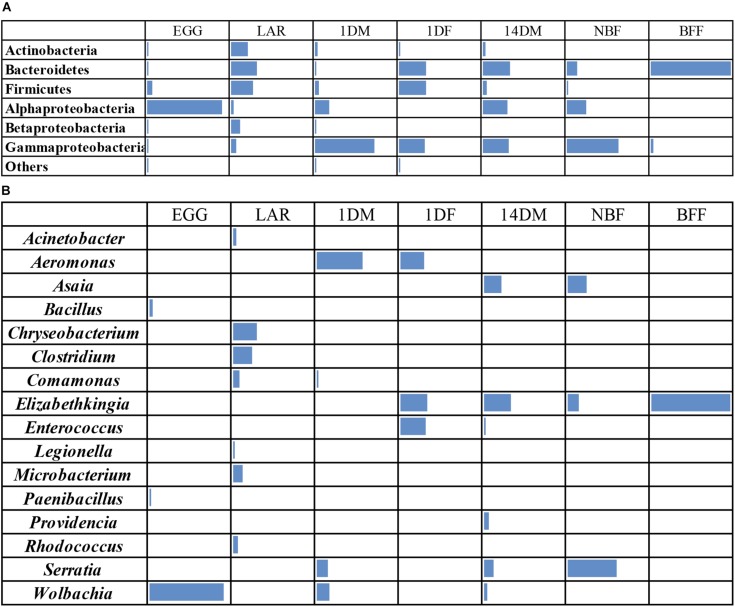
Heatmaps of bacterial taxa identified by 16S *rRNA* gene sequence analysis in *Aedes albopictus* eggs (EGG), guts of larvae (LAR), and guts of 1-day old teneral males (1DM), 1-day old teneral females (1DF), 14-day old sugar-fed males (14DM), 14-day old sugar-fed females (NBF) and 14-day old blood-fed females (BFF). Taxa were grouped at **(A)** phylum level, except Proteobacteria that were grouped at the class level and **(B)** genus level. Cells with a RA less than 1% they appear empty.

Our amplicon sequence analysis indicated that the EGG samples were dominated by Alphaproteobacteria (89.5%), mainly by *Wolbachia*, followed by Firmicutes and Bacteroidetes at 5.8% and 1% respectively (Figures 2A,B). In LAR samples, bacterial diversity was high and was evenly distributed between Bacteroidetes (31%), Firmicutes (28.8%), and Actinobacteria (20.8%), followed by Betaproteobacteria (10.6%), Gammaproteobacteria (6.8%), and Alphaproteobacteria (3.3%) (Figure 2A). The most dominant genera were those of *Chryseobacterium*, and *Clostridium* and to a lesser degree *Microbacterium*, *Comamonas*, *Rhodococcus*, and *Acinetobacter* (Figure 2B). The 1DM samples were dominated by Gammaproteobacteria (72%) followed by Alphaproteobacteria (16.6%), Firmicutes (4.3%), Actinobacteria (3%), Betaproteobacteria (2.2%), and Bacteroidetes (1%), while the 1DF samples were displaying a more even distribution between Gammaproteobacteria (31.1%), Firmicutes (32.2%), and Bacteroidetes (33.2%) (Figure 2A). This clear differentiation was also reflected at the genus level. The 1DM samples were dominated by *Aeromonas* sp. followed by *Wolbachia* and *Serratia* sp., while the 1DF samples were dominated by *Elizabethkingia* sp., *Enterococcus* sp. and *Aeromonas* sp. (Figure 2B). The 14DM samples, the bacterial diversity was evenly distributed between Gammaproteobacteria (30.4%), Alphaproteobacteria (28.8%), and Bacteroidetes (32.5%), followed by Firmicutes (5.1%) and Actinobacteria (2.6%) while the NBF exhibited a similar diversity but with the Gammaproteobacteria being abundant (62.4%) followed by Alphaproteobactreria (22.6%), and Bacteroidetes (13.1%) (Figure 2A). Interestingly, the BFF were almost exclusively dominated by Bacteroidetes (96.4%) (Figure 2A). At the genus level, the 14DM samples were characterized by the presence of *Elizabethkingia* sp. followed by *Asaia* sp., *Serratia* sp., *Enterobacter* sp., *Providencia* sp., and *Wolbachia* sp., the NBF samples were dominated by *Serratia* sp. and *Asaia* sp. followed by *Elizabethkingia* sp., while the BFF samples were dominated by *Elizabethkingia* sp., (95.4%) and *Chryseobacterium* sp. (0.94%) (Figure 2B).

**FIGURE 3:**
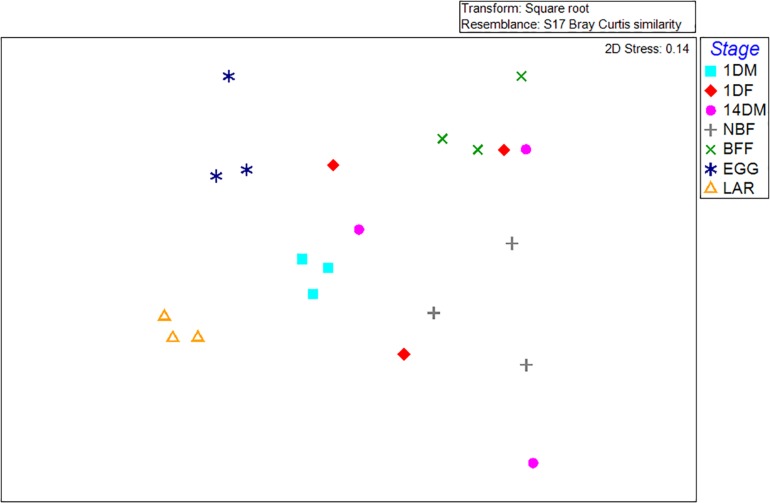
Non-metric multidimensional scaling (MDS) plot based on Bray–Curtis dissimilarities visualizes differences in bacterial community structures according to age, developmental stage, sex and diet. Orange triangles inverted blue and cyan squares, red diamonds, pink circles, gray crosses, and green multiplication sign represent *Aedes albopictus* eggs (EGG), guts of larvae (LAR), and guts of 1-day old teneral males (1DM), 1-day old teneral females (1DF), 14-day old sugar-fed males (14DM), 14-day old sugar-fed females (NBF) and 14-day old blood-fed females (BFF), respectively.

**FIGURE 4:**
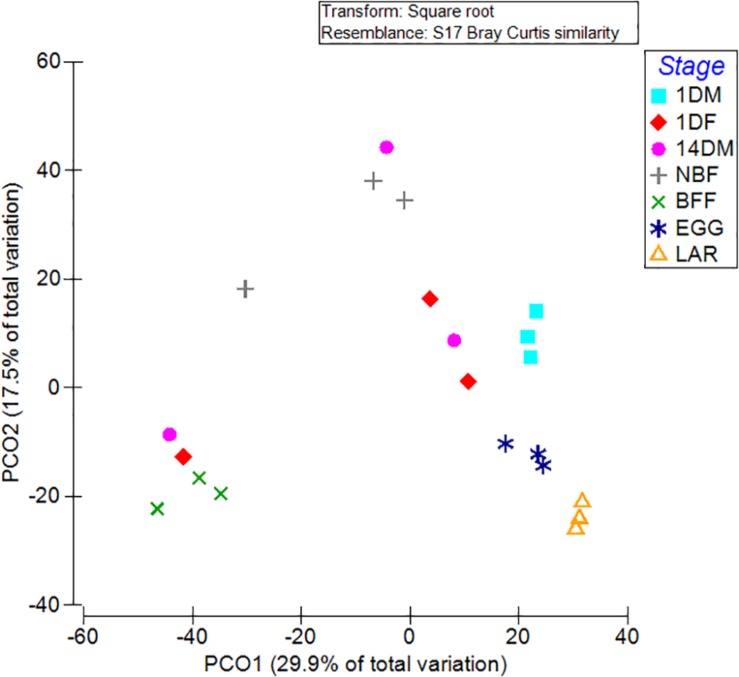
Principal coordinate analysis (PC*o*A) of bacterial communities based on the relative abundance of OTUs with ordinations from *Aedes albopictus* eggs (EGG), guts of larvae (LAR), and guts of 1-day old teneral males (1DM), 1-day old teneral females (1DF), 14-day old sugar-fed males (14DM), 14-day old sugar-fed females (NBF) and 14-day old blood-fed females (BFF). Variance explained by each PC*o*A axis is given in parentheses, while the main taxa that affect ordination clustering is presented.

Non-metric multi-dimensional scaling (MDS) and principal coordinates analysis (PCoA) indicated that the samples examined were clustered mainly based on the developmental stage and the diet used (Figures 3, 4). In more detail, the clusters between eggs and larvae were statistically significant (PERMANOVA, *P* < 0.001), as were those between the guts of 1-day and 14-day old adults (PERMANOVA, *P* < 0.001). Interestingly, there was a statistically significant difference between the gut samples of 14-day old *Ae. albopictus* females fed with sucrose (NBF) or blood (BFF) (PERMANOVA, *P* < 0.001).

### Relative Density of Selected Bacterial Taxa During *Aedes albopictus* Development

**FIGURE 5:**
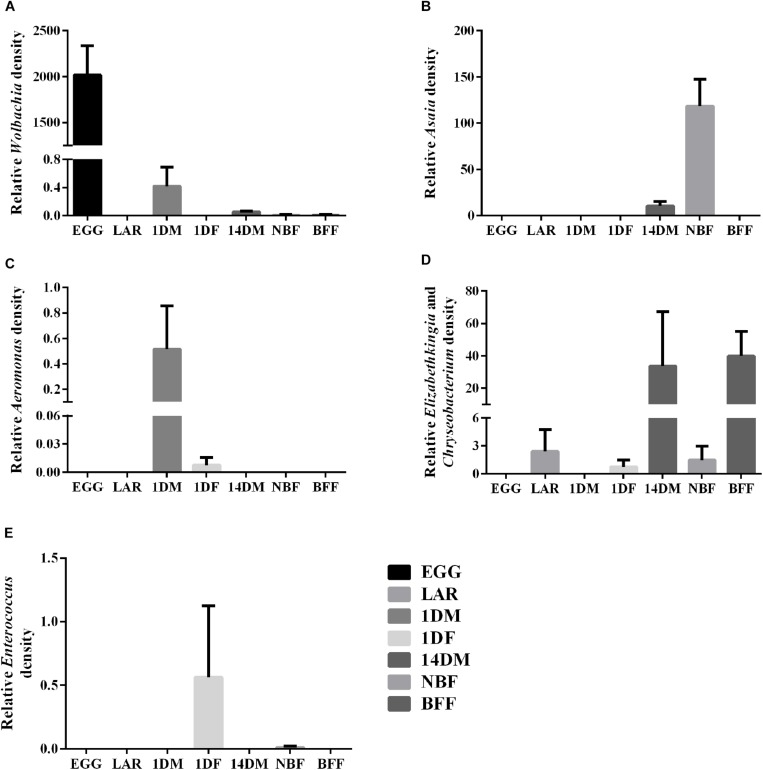
Relative abundance of five bacterial groups based on qPCR. **(A)** *Wolbachia*; **(B)** *Asaia*; **(C)** *Aeromonas*; **(D)** *Elizabethkingia*-*Chryseobacterium* group; **(E)** *Enterococcus.*

Based on the NGS data, we selected some of the most abundant bacterial taxa, *Aeromonas, Asaia*, *Chryseobacterium-Elizabethkingia* groups, *Enterococcus* and *Wolbachia*, to investigate their relative density by qPCR (Figure 5 and Supplementary Table 3). The data clearly indicated that: (a) *Wolbachia* was dominant in the EGG samples and it could also be detected at lower densities in 1DM, 14DM, NBF and BFF samples (Tukey HSD, *P < 0.0001*); (b) *Asaia* was detected at higher densities in the NBF samples and at much lower densities in 14DM samples (Tukey HSD, *P < 0.0001*); (c) *Aeromonas* was detected in the 1DM samples and in one of the biological replicates of the 1DF samples (Tukey HSD, *P = 0.0950*); (d) the *Elizabethkingia–Chryseobacterium* group was present in high densities in BFF samples, and it was also detected in some biological replicates of the LAR, 1DF, 14DM as well as in the NBF samples; however, there was no statistically significant difference among the different groups (Tukey HSD, *P = 0.0496*). It is worth noting that, based on the NGS data, *Elizabethkingia-Chryseobacterium* was only detected in BFF, NBF, 14DM, 1DF but not in LAR, and (e) *Enterococcus* was detected at higher densities in one replicate of the 1DF samples and at lower densities in one replicate of the NBF samples (Tukey HSD, *P* = 0.0950).

## Discussion

By employing a cultivation-dependent and a cultivation-independent approach, significant information on the composition of gut-associated microbiota of lab-reared *Ae. albopictus* along developmental stages, sex and feeding regimes was obtained. Although our study provides useful information for a colonized population of an SIT targeted species, the results can not be generalized. However, this information can be used to improve the effectiveness of mosquito population control strategies such as SIT, IIT and others through, for example, the assessment of cultivable bacteria as potential probiotics to enhance the rearing efficiency and quality of mass-produced insects.

### The Composition of Gut-Associated Microbiota of Lab-Reared *Aedes albopictus*

The cultivation-dependent approach resulted in the isolation of mosquito-associated bacteria which were assigned to 13 different genera (Table 1). The majority of them were Gram-negative, and mainly Gammaproteobacteria of the Enterobacteriaceae family, which have been previously described in other mosquito species such as *Ae. triseriatus*, *An. albimanus*, *An. gambiae*, *An. stephensi*, *C. pipiens*, *C. quinquefasciatus*, and *C. tarsalis* (Chao and Wistreich, 1959; Demaio et al., 1996; Pumpuni et al., 1996; Straif et al., 1998; Fouda et al., 2001; Gonzalez-Ceron et al., 2003; Pidiyar et al., 2004; Lindh et al., 2005), as well as in *Ae. albopictus* (Zouache et al., 2011; Otta et al., 2012; Minard et al., 2013b, 2014; Valiente Moro et al., 2013; Yadav et al., 2015), suggesting that they are widespread and constantly associated with mosquitoes.

Some gut-associated bacteria genera detected in the present study have been related with host’s biological process. *Acinetobacter* play a role in both blood digestion and nectar assimilation of *Ae. albopictus* (Minard et al., 2013b). *Asaia* may provide *An. stephensi* with vitamins (Crotti et al., 2010). *Asaia* and *Elizabethkingia* have been considered as candidates in paratransgenic approaches for mosquito control (Favia et al., 2007; Chen et al., 2015). *Serratia* and *Enterobacter* contain hemolytic enzymes and play a role in blood digestion of *Ae. aegypti* (Gaio et al., 2011). *Serratia* also play a role in chitin degradation of puparium (Iverson et al., 1984). However, *S. marcescens* was reported as pathogen of artificial-reared insects (Grimont and Grimont, 2015). As strains of *Enterobacter* have been proved candidates of probiotic for the Mediterranean fruit fly (Augustinos et al., 2015), isolation of these bacteria from *Ae. albopictus* provide potential material for further probiotic studying. *Asaia* played a specific role in the larval development of *An. stephensi* and reduced the developmental time before pupation (Crotti et al., 2010). It’s also worth noting that *Cedecea* spp. have been reported in the midgut of *Psorophora columbiae* (Demaio et al., 1996) and both field-collected and lab-reared *An. gambiae* (Pumpuni et al., 1996; Straif et al., 1998) while *Cedecea* spp. was also detected in the midgut of *Ae. aegypti* females (Gusmao et al., 2010). Interestingly, we isolated *Cedecea* spp. from larval guts as well from the egg surface of lab-reared *Ae. albopictus* suggesting a potential route for their inter-stadial transmission and these isolates may also represent potential probiotic candidates.

Previous studies in *Anopheles* showed lower diversity of gut-associated bacteria in lab-reared mosquitoes than wild populations (Gonzalez-Ceron et al., 2003; Rani et al., 2009). *Klebsiella*, which has been proven to be an effective probiotic of the Mediterranean fruit fly (Ben Ami et al., 2010; Gavriel et al., 2011; Kyritsis et al., 2017), has also been reported as a component of gut-associated bacterial communities in mosquitoes (Chao and Wistreich, 1960; Demaio et al., 1996; Straif et al., 1998; Terenius et al., 2012) including wild populations of *Ae. albopictus* (Crotti et al., 2009; Gusmao et al., 2010; Zouache et al., 2011; Valiente Moro et al., 2013; Yadav et al., 2015). However, this group was not detected in our study through both the cultivation-dependent and the cultivation-independent assays. Since there are documented differences among populations (Demaio et al., 1996; Zouache et al., 2011), this could be attributed to its absence from the original wild population. However, it could also be a result of a domestication process and the continuous artificial rearing. Similar to genetic changes, symbiotic changes may happen due to phenomena such as bottlenecks and small effective population size. Moreover, symbionts that are not crucial in the new environment may disappear and new may emerge, depending on the new rearing conditions.

Some of the bacterial isolates, such as *Enterobacter*, *Cedecea* (Enterobacteriaceae), *Stenotrophomonas* (Xanthomonadaceae), *Pseudomonas* (Pseudomonadaceae), and *Staphylococcus* (Staphylococcaceae) were not very abundant groups in NGS. It is well known that many factors including selectivity of culture-medium for specific bacterial taxa, growth rate and small colony number bias could influence the isolation process (Jannasch and Jones, 1959; Amann et al., 1995). NGS based culture-independent methods give relatively complete, albeit semi-quantitative, profile of the bacteria community. However, bias of Illumina could be induced too, for example, by PCR amplification protocols, primer choice and short read lengths (Claesson et al., 2010; Schloss et al., 2011; Pinto and Raskin, 2012; Tremblay et al., 2015; Gohl et al., 2016). It is interesting to note, however, that our NGS study provided similar Shannon diversity indices with those of previous studies which investigated either laboratory populations or populations of recent invasions and clearly lower when compared with that observed in established wild populations (Coon et al., 2016b; Hegde et al., 2018; Rosso et al., 2018). The bacterial profiles including the *Aeromonas* and *Serratia* co-occurrence pattern detected in the present study have also been previously observed (Hegde et al., 2018).

### Impact of Sex, Age and Diet on Gut-Associated Bacteria

Our study clearly shows that there are differences between males and females, and age and diet may also contribute to these differences. Clustering analysis suggested that the effect of diet was more significant than that of sex. After the full digestion of blood meal, bacteria diversity decreased significantly in accordance with previous study in *An. gambiae* (Wang et al., 2011). Over 95% of the gut-associated bacteria were *Elizabethkingia* and *Chryseobacterium*, mostly the former one. This closely related group may possess a competitive advantage over the other bacteria. *Elizabethkingia* was dominant in the guts of both sugar-fed and blood-fed females (Wang et al., 2011). It has been reported that *Elizabethkingia anophelis* could contribute nutrients by participating in erythrocyte lysis in the mosquito midgut (Chen et al., 2015).

Among cultivable strains, *Serratia* could not be isolated after blood feeding, in contrast to *Enterobacter* and *Pseudomonas*. These genera have also been reported in field-caught female *Anopheles* and *Culex* mosquitoes (Gonzalez-Ceron et al., 2003; Pidiyar et al., 2004; Lindh et al., 2005; Rani et al., 2009; Chavshin et al., 2012). In wild-caught *An. stephensi*, *Cryseobacterium*, *Pseudomonas* and *Serratia* were identified only in females (Rani et al., 2009). Our results indicated that *Pseudomonas* and *Enterobacter* may be enhanced with blood meal in *Ae. albopictus*, and a similar observation has been made in *An. gambiae* (Wang et al., 2011). On the other hand, *Serratia* were relatively stable during the adult stage of *Ae. albopictus* females while in *An. gambiae*, it increased after blood meal and decreased later (Wang et al., 2011). Interestingly, *Enterobacter* sp. and *Serratia* sp. showed strong hemolytic activity among bacteria isolated from *Ae. aegypti* midgut while *Serratia* was found to be dominant in all isolations during blood digestion in the same species (Gaio et al., 2011) (Gusmao et al., 2010). It’s also worth noting that Azambuja and colleagues (Azambuja et al., 2004) isolated a *S. marcescens* strain which was able to lyse erythrocytes from guts of blood feeding *Rhodnius prolixus*. The nutrient composition of food sources was considered an explanation of the differential bacterial population structure between sexes (Minard et al., 2013b). Our results suggested that there was a strong effect of adult diet, like blood meal, on the structure of gut-associated bacterial communities.

Our study indicated that age also plays an important role in shaping the bacterial communities. For example, *Wolbachia* and *Aeromonas* were found at high densities in 1-day old males but their densities were drastically decreased in older males. On the other hand, the *Elizabethkingia-Chryseobacterium* group was essentially absent in young males and drastically increased in 14-days old males.

### Dynamics and *Trans*-Stadial Transmission of Gut-Associated Bacteria Under Lab-Rearing Conditions

The present study identified several genera shared among control groups and mosquito samples. The isolates from larval food and sugar solution belonged to Acinetobacter, Bacillus, Enterobacter, Pseudomonas and Raoultella bacterial genera. In previous studies, *Enterobacter* sp. was shown to immigrate successfully in both larval and adult stages, *Bacillus* sp. to settle through food into larval guts whereas *Raoultella* sp. and *Pseudomonas* sp. to fail to settle in larval guts or to be maintained in a detectable level. In addition, the *Acinetobacter* sp. was found not only in mosquito guts but also in breeding sites and food sources (reviewed in Minard et al., 2013b).

Our study also provided a view of the bacterial dynamic among life stages of *Ae. albopictus* clearly indicating that gut-associated bacteria diversity changed significantly between eggs and larvae, teneral and 14-day old adults. Food (larval food, sugar solution, blood) is certainly a contributing factor for these changes and the transmission of some of these bacteria. *Wolbachia, Asaia*, and *Elizabethkingia* have been shown to pass on to eggs from parents (Kittayapong et al., 2002; Favia et al., 2007; Akhouayri et al., 2013). Gusmao and colleagues (Gusmao et al., 2010) identified *Asaia* sp. and *Enterobacter* sp. from *Ae. aegypti* eggs and considered them to be probably transovarially transmitted. Based on our NGS data, it was shown that *Ae. albopictus* eggs contained bacteria from more than 15 genera including *Bacillus*, *Chryseobacterium* and *Escherichia-Shigella* which were among the main groups in female adults both before and after blood feeding. *Cedecea* sp. and *Elizabethkingia* sp. were isolated from *Ae. albopictus* eggs and detected outside the eggs, suggesting that they are transmitted vertically via “egg smearing.” Akhouayri et al. (2013) externally treated embryos of *An. gambiae* with antiseptic solution and dramatically reduced the melanotic pathology which was caused by *Elizabethkingia meningoseptica* suggesting that it could be transmitted to embryos via “egg smearing.” In *An. gambiae*, one of the transmission routes of *Asaia* was also reported to be “egg smearing” (Damiani et al., 2010). Further studies are necessary to get knowledge on how these taxa are transmitted.

In conclusion, using culture-dependent and culture-independent approaches, we characterized mainly the microbiota associated with a laboratory strain of *Ae. albopictus*, which is reared under artificial rearing conditions for potential SIT-based applications, throughout the mosquito developmental stages (egg, larva and adult), at different adult ages (both young and old males and females) and female feeding regimes (sugar or blood). It is worth noting that a relatively high diversity of bacteria was detected in eggs (a stage understudied in mosquitoes), some of which were also found in adult females. Overall, our study clearly shows that developmental stage and diet are the key factors shaping the microbiota. The density levels of some of the most abundant taxa (*Aeromonas, Asaia*, *Chryseobacterium-Elizabethkingia* groups, *Enterococcus*, and *Wolbachia*) in different developmental stages and diets were determined by PCR. However, this should in the future be extended to other abundant bacterial taxa such as *Serratia*, *Clostridium*, and *Providencia*. Our study identified several species which may worth be investigated about their potential probiotic properties including members of the Enterobacteriaceae (*Aeromonas*, *Elisabethkingia*, *Enterococcus*, *Enterobacter*, *Providencia*) and Acetobacteriaceae (*Asaia*) families to enhance production and improve quality of mass-reared mosquito species which are to be used for SIT and other related population suppression programs.

## Data Availability Statement

The datasets generated for this study can be found in the 16S rRNA gene sequences reported in this study have been deposited in NCBI under Bioproject number PRJNA575054, while the Sanger generated sequences have been deposited under accession numbers MN540103 to MN540125.

## Author Contributions

SC performed the experiments and drafted the manuscript. DZ performed the experiments, analyzed the data, and drafted the manuscript. AA performed the experiments and critically revised the manuscript. VD and NB performed the bioinformatic analysis and critically revised the manuscript. GT performed the bioinformatic analysis, interpreted the data, contributed to the drafting and critical revision of the manuscript. HM performed gut samples dissection and collection and critically revised the manuscript. KB conceived the study, designed the experiments, interpreted the data, contributed to the drafting, and critically revised the manuscript. All authors approved the final version of the manuscript and agreed to be accountable for all aspects of the work in ensuring that questions related to the accuracy or integrity of any part of the work are appropriately investigated and resolved.

## Conflict of Interest

The authors declare that the research was conducted in the absence of any commercial or financial relationships that could be construed as a potential conflict of interest.

## Footnotes

1 http://www2.decipher.codes/index.html

2 http://fungene.cme.msu.edu/FunGenePipeline/chimera_check/form.spr

3 http://www.ncbi.nlm.nih.gov/BLAST/

4 http://rdp.cme.msu.edu/classifier/classifier.jsp
