# Supplementary material for: Multiple Factors Determine the Structure of Bacterial Communities Associated With Aedes albopictus Under Artificial Rearing Conditions
Source: Front Microbiol. 2020 Apr 15;11:605. doi: 10.3389/fmicb.2020.00605 (PMC7176356; doi:10.3389/fmicb.2020.00605)
Supplement: Supplementary file 1 [file Data_Sheet_1.docx]

**Supplementary Table 1:** Primers used in the present study

| **Target taxon** | **Primer** | **Sequence (5'-3')** | **T(A)** | **Reference** |
| --- | --- | --- | --- | --- |
| *Enterococcus* | Entbes-3 | TTGCTAGAGTGCCCAACTGAAT | 66 | This study |
|  | Entbes-5 | CCCTTACCAGGTCTTGACATCC |  |  |
| *Aeromonas* | IA-F | CTGAACCAGAACAAGACCCCG | 60 | (Khan et al., 2009) |
|  | IA-R | ATGTTGTTGGTGAAGCAGTA |  |  |
| *Asaia* | Asa-For | GCGCGTAGGCGGTTTACAC | 60 | (Chouaia et al., 2012) |
|  | Asa-Rev | AGCGTCAGTAATGAGCCAGGTT |  |  |
| *Wolbachia* | wsp-F | ATCTTTTATAGCTGGTGGTGGT | 58 | (Hussain et al., 2011) |
|  | wsp-R | GGAGTGATAGGCATATCTTCAAT |  |  |
| *Chryseobacterium-Elizabethkingia* group | CEP3 | GAAAGCGTGGGGAGCGAACA | 58 | This study |
|  | CEP8 | CGACAACCATGCAGCACCTTG |  |  |
| *Aedes albopictus* (housekeeping gene) | Rps6-qpcrF | CGTCGTCAGGAACGTATTCG | 58 | (Zheng et al., 2019) |
|  | Rps6-qpcrR | TCTTGGCAGCCTTGACAGC |  |  |

**References**

Chouaia, B., Rossi, P., Epis, S., Mosca, M., Ricci, I., Damiani, C., et al. (2012). Delayed larval development in *Anopheles* mosquitoes deprived of *Asaia* bacterial symbionts. *BMC Microbiol* 12 Suppl 1, S2. doi:10.1186/1471-2180-12-S1-S2.

Hussain, M., Frentiu, F. D., Moreira, L. A., O’Neill, S. L., and Asgari, S. (2011). *Wolbachia* uses host microRNAs to manipulate host gene expression and facilitate colonization of the dengue vector *Aedes* *aegypti*. *Proceedings of the National Academy of Sciences of the United States of America* 108, 9250–9255.

Khan, I. U., Loughborough, A., and Edge, T. A. (2009). DNA-based real-time detection and quantification of aeromonads from fresh water beaches on Lake Ontario. *J Water Health.* 7, 312–323.

Zheng, X., Zhang, D., Li, Y., Yang, C., Wu, Y., Liang, X., et al. (2019). Incompatible and sterile insect techniques combined eliminate mosquitoes. *Nature* 572, 56–61. doi:10.1038/s41586-019-1407-9.

**Supplementary Table 2** PCR-RFLP profiling of the bacterial strains isolated

| **Isolate No.** | ***Taq*I** | ***Eco*RI** | ***Hae*III** | ***Rsa*I** |
| --- | --- | --- | --- | --- |
| 1_150 | 573, 360, 185, 170, 86, 53, 35 | 817, 645 | 317, 210, 204, 180, 165, 161, 104, 68, 34, 19 | 502, 457, 405, 98 |
| 2_71 | 413, 360, 347, 170, 86, 53, 35 | 817, 647 | 317, 210, 204, 180, 167, 161, 104, 68, 34, 19 | 502, 457, 405, 98 |
| 3_272 | 411, 360, 185, 170, 162, 86, 53, 35 | 817, 645 | 317, 210, 204, 180, 165, 161, 104, 68, 34, 19 | 502, 457, 405, 98 |
| 4_48 | 573, 360, 185, 170, 86, 53, 35 | 817, 645 | 317, 210, 204, 180, 165, 161, 104, 68, 34, 19 | 502, 457, 405, 98 |
| 5_149 | 758, 360, 170, 86, 53, 35 | 817, 645 | 317, 278, 204, 180, 165, 161, 104, 34, 19 | 502, 457, 405, 98 |
| 6_380 | 760, 360, 170, 86, 53, 35 | 817, 647 | 317, 210, 204, 186, 180, 161, 104, 68, 34 | 864, 502, 98 |
| 7_336 | 758, 361, 170, 86, 53, 35 | 818, 645 | 317, 279, 204, 180, 165, 161, 104, 34, 19 | 862, 401, 102, 98 |
| 8_200 | 682, 361, 170, 86, 80, 53, 35 | 818, 649 | 317, 220, 204, 171, 169, 161, 104, 59, 34, 19, 9 | 866, 357, 146, 98 |
| 9_139 | 804, 361, 170, 89, 35 | 1459 | 682, 220, 171, 161, 104, 59, 34, 19, 9 | 624, 357, 234, 146, 98 |
| 10_230 | 483, 360, 324, 170, 89, 35 | 817, 644 | 326, 278, 235, 187, 180, 89, 85, 66, 15 | 732, 356, 146, 129, 98 |
| 11_158 | 483, 360, 324, 170, 89, 35 | 817, 644 | 326, 278, 235, 187, 180, 89, 85, 66, 15 | 424, 356, 308, 146, 129, 98 |
| 12_85 | 481, 361, 324, 170, 89, 35 | 818, 642 | 326, 233, 220, 171, 96, 91, 89, 85, 66, 59, 15, 9 | 859, 357, 146, 98 |
| 13_119 | 795, 284, 171, 105, 39, 38, 35 | 820, 647 | 513, 220, 220, 172, 151, 89, 59, 19, 15, 9 | 459, 406, 342, 162, 98 |
| 14_229 | 803, 293, 170, 87, 67, 35 | 816, 639 | 326, 312, 284, 178, 151, 119, 85 | 407, 404, 356, 146, 98, 44 |
| 15_124 | 803, 289, 170, 89, 67, 35 | 812, 641 | 326, 308, 284, 151, 123, 100, 85, 57, 19 | 409, 404, 352, 146, 98, 44 |
| 16_39 | 907, 361, 146, 35, 26 | 821, 654 | 1185, 290 | 503, 466, 406, 100 |
| 17_468 | 771, 530, 138, 35 | 818, 656 | 565, 457, 291, 105, 34, 22 | 468, 406, 355, 146, 99 |
| 18_74 | 797, 318, 164, 123, 37 | 1439 | 504, 274, 241, 145, 124, 97, 20, 19, 15 | 402, 352, 214, 161, 146, 73, 57, 34 |
| 19_244 | 465, 332, 318, 164, 123, 37 | 1439 | 504, 274, 241, 145, 124, 97, 20, 19, 15 | 402, 352, 214, 161, 146, 73, 57, 34 |
| 20_287 | 465, 332, 318, 164, 123, 37 | 1439 | 504, 274, 241, 145, 124, 97, 20, 19, 15 | 402, 352, 214, 161, 146, 73, 57, 34 |
| 21_129 | 804, 361, 170, 89, 35 | 1459 | 682, 220, 171, 161, 104, 59, 34, 19, 9 | 624, 357, 234, 146, 98 |
| 22_97 | 682, 361, 170, 86, 80, 53, 35 | 818,649 | 317, 220, 204, 171, 169, 161, 104, 59, 34, 19, 9 | 866, 357, 146, 98 |
| 23_363 | 893, 361, 170, 34 | 1458 | 682, 220, 171, 160, 104, 59, 34, 19, 9 | 857, 357, 146, 98 |

**Supplementary Table 3** qPCR statistical analysis

| Biological set | n | Mean±SE/Tukey HSD | ANOVA |
| --- | --- | --- | --- |
| *Wolbachia* |  |  | F(3,20)=40.1715, P<0.0001 |
| egg | 3 | 2018.67±318.49, A(P<0.0001) |  |
| larvae | 3 | 0.00±0.00, B |  |
| 1dmale | 3 | 0.42±0.27, B |  |
| 1dfemale | 3 | 0.00±0.00, B |  |
| 14dmale | 3 | 0.05±0.02, B |  |
| 14dfemale | 3 | 0.01±0.01, B |  |
| BFfemale | 3 | 0.01±0.01, B |  |
|  |  |  |  |
| *Asaia* |  |  | F(6,19)=15.0910, P<0.0001 |
| EGG | 3 | 0.00±0.00, B |  |
| LAR | 3 | 0.00±0.00, B |  |
| 1DM | 3 | 0.00±0.00, B |  |
| 1DF | 3 | 0.00±0.00, B |  |
| 14DM | 2 | 10.54±4.75, B |  |
| NBF | 3 | 118.59±28.94, A(P<0.0001) |  |
| BFF | 3 | 0.00±0.00, B |  |
|  |  |  |  |
| *Aeromonas* |  |  | F(6,20)=2.2864, P=0.0950 |
| EGG | 3 | 0.00±0.00, A |  |
| LAR | 3 | 0.00±0.00, A |  |
| 1DM | 3 | 0.51±0.34, A |  |
| 1DF | 3 | 0.01±0.01, A |  |
| 14DM | 3 | 0.00±0.00, A |  |
| NBF | 3 | 0.00±0.00, A |  |
| BFF | 3 | 0.00±0.00, A |  |
|  |  |  |  |
| *Chryseobacterium-Elizabethkingia* group |  |  | F(6,19)=2.9239, P=0.0496 |
| EGG | 3 | 0.00±0.00, A |  |
| LAR | 3 | 2.41±2.33, A |  |
| 1DM | 3 | 0.00±0.00, A |  |
| 1DF | 3 | 0.74±0.74, A |  |
| 14DM | 2 | 33.61±33.61, A |  |
| NBF | 3 | 1.47±1.47, A |  |
| BFF | 3 | 39.75±15.33, A |  |
|  |  |  |  |
| *Enterococcus* |  |  | F(6,20)=0.9938, P=0.4663 |
| EGG | 3 | 0.00±0.00, A |  |
| LAR | 3 | 0.00±0.00, A |  |
| 1DM | 3 | 0.00±0.00, A |  |
| 1DF | 3 | 0.56±0.56, A |  |
| 14DM | 3 | 0.00±0.00, A |  |
| NBF | 3 | 0.01±0.01, A |  |
| BFF | 3 | 0.00±0.00, A |  |

**Supplementary Figure Legends**

**Suppl. Figure 1** Simpson, Shannon and Pielou diversity indices for the two hypervariable regions examined. Boxes denote the interquartile range (IQR), the line within the box is the median, and whiskers indicate 1.5xIQR. The black dots represent outliers.

**Suppl. Figure 2** Diversity of the gut symbiotic community of the two hypervariable regions examined at phylum level.

**Suppl. Figure 3** Diversity of the gut symbiotic community of the two hypervariable regions examined at class level.

**Suppl. Figure 4** Diversity of the gut symbiotic community of the two hypervariable regions examined at family level.

**Suppl. Figure 5** Rarefaction curves

**Suppl. Figure 1**

**Suppl. Figure 2**

**Suppl. Figure 3**

**Suppl. Figure 4**

**
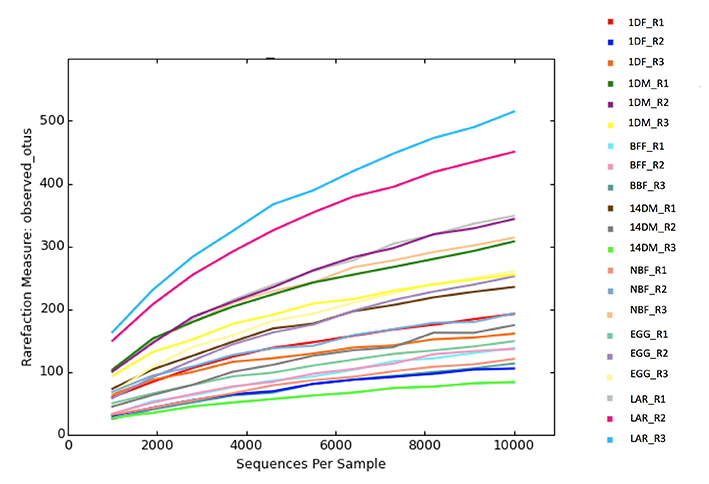
**

**Suppl. Figure 5**
